# In-depth single-cell and bulk-RNA sequencing developed a NETosis-related gene signature affects non-small-cell lung cancer prognosis and tumor microenvironment: results from over 3,000 patients

**DOI:** 10.3389/fonc.2023.1282335

**Published:** 2023-10-19

**Authors:** Liangyu Zhang, Xun Zhang, Maohao Guan, Fengqiang Yu, Fancai Lai

**Affiliations:** ^1^ Department of Thoracic Surgery, the First Affiliated Hospital, Fujian Medical University, Fuzhou, China; ^2^ Department of Thoracic Surgery, National Regional Medical Center, the First Affiliated Hospital, Fujian Medical University, Fuzhou, China

**Keywords:** NEtosis, non-small-cell lung cancer, immune, single-cell, prognosis biomarker

## Abstract

**Background:**

Cell death caused by neutrophil extracellular traps (NETs) is known as NETosis. Despite the increasing importance of NETosis in cancer diagnosis and treatment, its role in Non-Small-Cell Lung Cancer (NSCLC) remains unclear.

**Methods:**

A total of 3298 NSCLC patients from different cohorts were included. The AUCell method was used to compute cells’ NETosis scores from single-cell RNA-sequencing data. DEGs in sc-RNA dataset were obtained by the Seurat’s “FindAllMarkers” function, and DEGs in bulk-RNA dataset were acquired by the DESeq2 package. ConsensusClusterPlus package was used to group patients into different NETosis subtypes, and the Enet algorithm was used to construct the NETosis-Related Riskscore (NETRS). Enrichment analyses were conducted using the GSVA and ClusterProfiler packages. Six distinct algorithms were utilized to evaluate patients’ immune cell infiltration level. Patients’ SNV and CNV data were analyzed by maftools and GISTIC2.0, respectively. Drug information was obtained from the GDSC1, and predicted by the Oncopredict package. Patient response to immunotherapy was evaluated by the TIDE algorithm in conjunction with the phs000452 immunotherapy cohort. Six NRGs’ differential expression was verified using qRT-PCR and immunohistochemistry.

**Results:**

Among all cell types, neutrophils had the highest AUCell score. By Intersecting the DEGs between high and low NETosis classes, DEGs between normal and LUAD tissues, and prognostic related genes, 61 prognostic related NRGs were identified. Based on the 61 NRGs, all LUAD patients can be divided into two clusters, showing different prognostic and TME characteristics. Enet regression identified the NETRS composed of 18 NRGs. NETRS significantly associated with LUAD patients’ clinical characteristics, and patients at different NETRS groups showed significant differences on prognosis, TME characteristics, immune-related molecules’ expression levels, gene mutation frequencies, response to immunotherapy, and drug sensitivity. Besides, NETRS was more powerful than 20 published gene signatures in predicting LUAD patients’ survival. Nine independent cohorts confirmed that NETRS is also valuable in predicting the prognosis of all NSCLC patients. Finally, six NRGs’ expression was confirmed using three independent datasets, qRT-PCR and immunohistochemistry.

**Conclusion:**

NETRS can serves as a valuable prognostic indicator for patients with NSCLC, providing insights into the tumor microenvironment and predicting the response to cancer therapy.

## Introduction

Human lung cancer is among the most deadly types of cancer and is associated with a high mortality rate and morbidity (1, 2). Lung cancer can be classified as SCLC (Small-Cell Lung Cancer) or NSCLC (Non-Small-Cell Lung Cancer). Among lung cancer cases, NSCLCs constitute the majority, and lung adenocarcinomas (LUADs) are the most common NSCLC type (3). The growth rate of LUAD is generally slower than that of squamous lung cancer (LUSC). However, it begins metastasizing earlier than LUSC (4). Molecular-targeted agents and immunotherapies are currently used to treat NSCLC, which are more effective and less harmful than traditional chemotherapy and radiotherapy (5). Although immunotherapy and targeted treatments offer advantages for a small proportion of patients, survival rates remain low. Consequently, finding reliable prognosticators for LUAD, or all NSCLC in general, is imperative.

NETs (Neutrophil Extracellular Traps) are histone and proteases-coated DNA structures released by neutrophils to trap microbes, and they are formed through a process known as NETosis (6, 7). NETs may play a role in non-infectious diseases, including autoimmune disease, coagulation disorders, acute injury, and cancer. There has also been research investigating its role in malignancies such as venous thromboembolism, invasive growth, and metastasis (8). Additionally, it’s also known that NETs increase tumor cells’ ability to metastasize within the bloodstream by enhancing the cell cycle (9). Researchers have demonstrated that NET-DNA, which is a component of NETs, promotes cancer metastasis through CCDC25, which is a transmembrane protein (10); and it has been shown that NETs formation triggers protumorigenic inflammatory responses and activates HCC metastasis (11). NETs are also reported to shield cancer cells from immune system’s attack and reduce immunotherapy’s efficacy (12, 13). Despite the growing importance of NETosis in cancer diagnosis and treatment, studies about its role in LUAD are limited.

Due to the importance of NETosis to LUAD, our research focused on developing a genetic pattern involving NETosis-associated genes. In the single-cell dataset, neutrophils scored highest for NETosis. Based on the NETosis score, all the cells were divided into two classes, and the class with higher NETosis had more active cell communication. Based on 61 prognostic related differently expressed NRGs, we identified two NETosis-related subtype, exhibited distinct prognosis and TME features. Then a novel NETosis-related Riskscore consist of eignteen NRGs was developed by the Enet algorithm. Significant variations were observed among LUAD patients in different NETRS categories in terms of immune cell infiltration, clinical features, prognosis, SNV and CNV variation frequencies, responsiveness to immunotherapy, and sensitivity to drug. By comparing NETRS with 20 published gene signatures, we found that NETRS was more powerful in predicting LUAD patients’ prognosis. Additionally, NETRS was able to predict the prognosis of patients with various types of NSCLC. The findings of this study suggest that NETosis may play a crucial role in developing therapeutic approaches for individuals diagnosed with LUAD, and could offer fresh perspectives and sources for future investigations into the function of NETosis in NSCLC.

## Materials and methods

### Data acquisition

Clinical information and bulk RNA sequencing data for LUAD and all NSCLC patients, and data on single nucleotide variations (SNV) and copy number variations (CNV) for LUAD patients, were downloaded from the TCGA website (https://portal.gdc.cancer.gov/) (14). SNV data was processed by the maftools package, and CNV data were analyzed using GISTIC2.0 (15). Ten GEO datasets for NSCLC patients, including GSE72094-LUAD, GSE31210-LUAD, GSE8894-NSCLC, GSE42127-NSCLC, GSE68465-NSCLC, GSE41271-NSCLC, GSE74777-NSCLC, 3141-NSCLC, GSE30219-NSCLC, and GSE37745-NSCLC, were obtained from the GEO database (https://www.ncbi.nlm.nih.gov/geo/) (16). The TCGA-NSCLC dataset comprises clinical and RNA sequencing information for LUAD and LUSC patients. The GSE72094 and GSE31210 datasets exclusively contain data for LUAD patients. Among the remaining eight GEO datasets, GSE74777 is the only one that solely includes data for LUSC patients, while the others all encompass data for patients with various types of NSCLC. These types include LUAD, LUSC, lung basal cell carcinoma, lung large cell carcinoma, and other NSCLC subtypes. It is important to note that the majority of these NSCLC subtypes are LUAD and LUSC. The GSE127465 dataset includes single-cell RNA-sequencing information for 7 primary LUAD samples. That data was downloaded from TISCH (17) and processed as described in previous (18). Gene sets for NETosis were compiled from previously published studies (**Table S1**) (19–21). Based on the AUCell method, the fraction of enrichment for NETosis-related gene expression was calculated in single cells (22). The TIDE score, which is an ICB response predictor for LUAD patients, was calculated on the TIDE website (http://tide.dfci.harvard.edu) (23), and an Immunotherapy cohort phs000452 was downloaded from the TIGER database (http://tiger.canceromics.org/#/) (24). From Thorsson V’s study (25), we acquired data on multi-omics, such as neoantigen load and aneuploidy.

### Cell - cell communication analysis

A comparison of intercellular communication frequencies and intensities between high and low networks was carried out in R-package ‘CellChat’ (26).

### Consensus clustering

Based on the expression of the 61 prognosis-related NETosis-Related Genes (NRGs), the R package ConsensusClusterPlus was used to effectively cluster the LUAD patients into different clusters. All LUAD patients could be clustered into two clusters, demonstrating prognostic and immunoinfiltration differences.

### Differential analysis

Using Seurat’s “FindAllMarkers” function, genes differentially expressed (DEGs) between two NETRS classes were identified, and genes had |log2 (fold change)| > 0.25 with adjusted *p*-value (*P*adj) <0.01 were considered as DEGs. DESeq2 package was used to identify DEGs between normal and LUAD tumor samples, and genes with *P*adj < 0.01 and |log2(Fold-change)|> 1 were included.

### Enrichment analysis

In order to understand the biological functions and potential signaling pathways associated with genes related to NETosis, GO and KEGG enrichment analysis were utilized. In order to uncover potential prognostic mechanisms related to NETRS, we performed enrichment analyses using GO, KEGG, and GSVA for genes with significant associations. GO and KEGG analyses were performed with the R package ‘ClusterProfiler’ (27), and GSVA was performed by ‘GSVA’ package (28). The sets of reference were named ‘c5.all.v7.0.symbols.gmt’.

### Immune infiltration analysis

A total of six algorithms were used to measure the extent of immune cell infiltration in the TCGA-LUAD dataset. TIMER, quantTIseq, MCP-counter, EPIC, and ESTIMATE algorithms are implemented using the R package ‘IOBR’ (29), while the ssGSEA algorithm is implemented by the R package “GSVA”. We also compared immune-related molecule expression between patients with high and low NETRS in the TCGA-LUAD dataset.

### Construction of the NETosis-related Riskscore

Genes associated with survival prognosis are identified through univariate Cox analysis, and NETRS was constructed through elastic network (Enet) algorithm. According to the C-Index, we adjust the α value in the Enet algorithm between 0.1 and 0.9, and finally determined 0.1 as the optimal α value. Based on the median NETRS value, patients with NSCLC were categorized into groups of high risk and low risk. PCA analysis was performed using the R package ‘status’, while the generation of time-dependent ROC curve was accomplished using the ‘survminer’ and ‘timeROC’ packages. In addition, through the R package ‘rms’, we construct a nomogram by combining NETRS with clinical factors. The calibration curve, time-ROC curve, and DCA curve were used to evaluate the nomogram.

### Predicting potential drugs target NETRS

The GDSC1 database (https://www.cancerrxgene.org/) (30) provides information about drug sensitive data and corresponding gene expression matrix. Based on the R package ‘oncoPredict’ (31), we calculated the IC50 value, chich is an indicator of drug sensitivity, for each sample.

### Validation of six NRGs’ expression

Sample Collection: Two datasets (GSE19188 and GSE43458) containing LUAD tissues and paracancerous tissues were downloaded from the GEO database. In addition, anatomical samples involving 8 pairs of LUAD and corresponding paracancerous tissues were collected from the First Affiliated Hospital of Fujian Medical University. All patients provided written informed consent, and the study protocol was approved by the Ethics Committee of the First Affiliate Hospital of Fujian Medical University. qRT-PCR analysis: Total RNA was extracted using an RNA extraction kit (Vazyme, China) following the manufacturer’s instructions. The extracted RNA was then reverse transcribed into cDNA using the All-in-One First-Strand Synthesis MasterMix kit (iScience, China). For qRT-qPCR analysis, triplicate aliquots of each cDNA sample were prepared using Taq SYBR® Green qPCR Premix (iScience, China). The internal reference gene used in this study was β-Actin. The primers of the six NRGs and the internal reference gene were shown in **Table S3**.

### Statistic analysis

R (version 4.1.1) was used for all statistical analysis. Using the Wilcoxon test or the t-test, the disparity between two groups was compared. In correlation analyses, Pearson or Spearman correlation coefficients were used. The K-M analysis was employed to forecast the disparity in overall survival between the low and high NETRS categories. Multivariate Cox regression analysis was conducted to examine the predictive significance of NETRS and clinicopathological features. If Ns- P is greater than or equal to 0.05, *- P is less than 0.05, **- P is less than 0.01, and ***- P is less than 0.001.

## Results

### Exploring NETosis-related cell type

**Figure 1:**
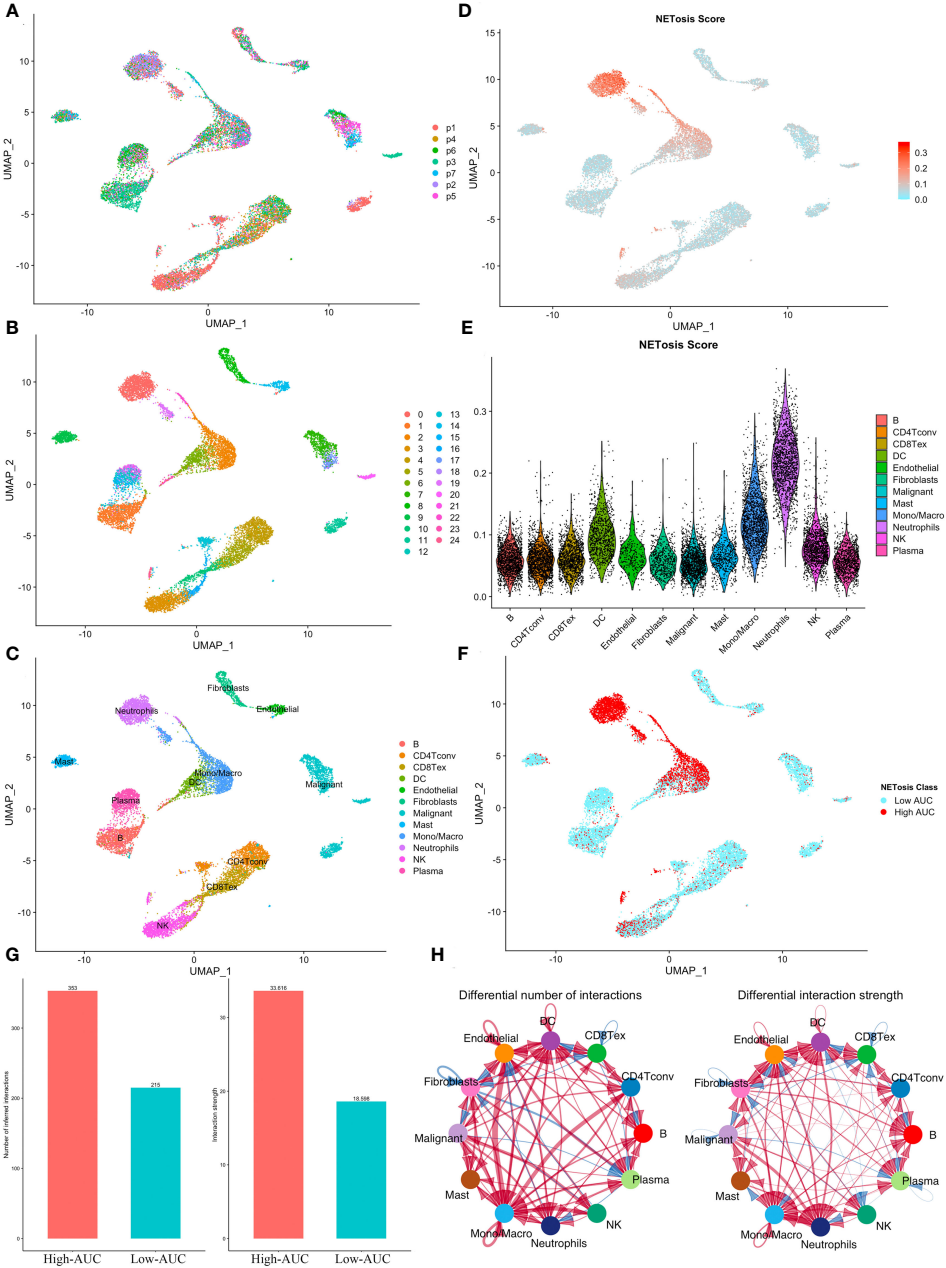
Identification of NETosis related active cells. **(A)** The UMAP plot shows the distribution of cells from 7 samples. **(B, C)** Cells were clustered into 25 clusters **(B)** and identified as 12 different types **(C)**. **(D)** The distribution of NETosis Score at the UMAP map. **(E)** Violin plot shows that neutrophils has highest NETosis Score. **(F)** Cells were classified into two clusters based on their NETosis score. **(G, H)** Comparison of the number and intensity of interaction between high and low NETosis Scores’ cells using the bar plot **(G)** and the network diagram **(H)**.

In the GSE127465 single-cell sequencing dataset, we obtained a total of 26,655 cells after initial quality control. Based on the UMAP map, we observed seven samples with a relatively uniform distribution of cells, indicating no obvious batch effects (**Figure 1A**). The cells were then clustered into 25 clusters (**Figure 1B**), and the meta-data from TISCH database was utilized to identify 12 cell types (**Figure 1C**). Further analysis using the AUCell algorithm revealed that neutrophils exhibited the highest NETosis Score (**Figures 1D, E**). Subsequently, we divided each cell population into two groups, High-AUC and Low-AUC, based on their mean NETosis Score (**Figure 1F**). Examination of cell-cell communication showed that cells with a high NETosis Score demonstrated more frequent and stronger communication compared to cells with a low NETosis score (**Figures 1G, H**).

### Consensus clustering identified two NETosis-related subtypes

**Figure 2:**
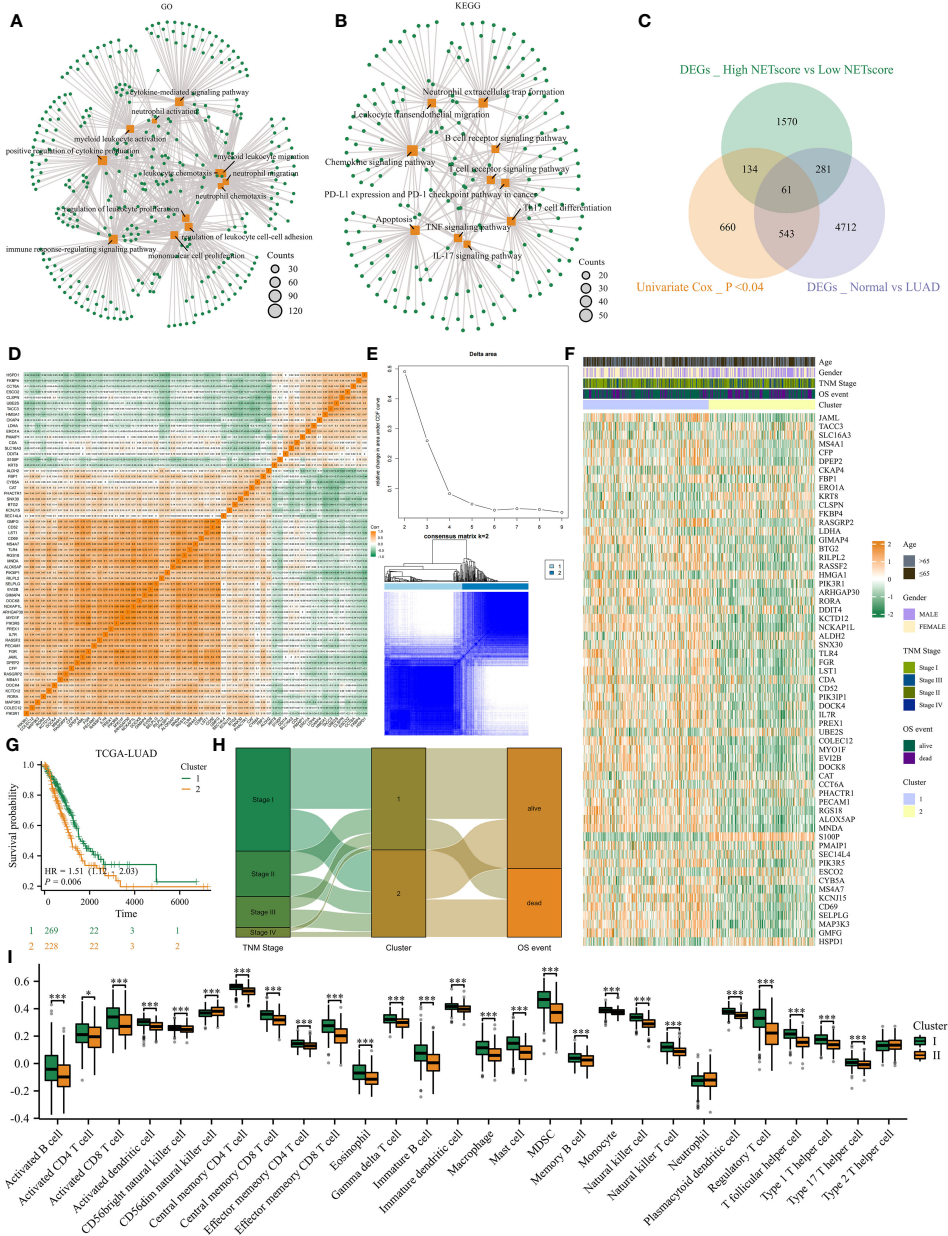
Consensus clustering identified two NETosis-related clusters**. (A, B)** GO **(A)** and KEGG **(B)** analysis on DEGs between high- and low- NETosis classes. **(C)** The Venn diagram. **(D)** The correlation heatmap illustrates the 61 genes’ relationship with each other. **(E)** When k=2, the cluster effect was best. **(F)** The 61 genes’ expression map between two clusters. **(G)** Two cluster patients’ different prognosis. **(H)** The sankey graph shows clusters’ association with clinical features. **(I)** Comparison of the immune infiltration level between cluster1 and cluster2, ‘*’ means P value is less than 0.05, ‘***’ means P value is less than 0.001.

With the help of the ‘FindAllMarkers’ function in the Seurat package, we identified differently expressed genes (DEGs) between high and low NETosis classes. GO analysis shows that these DEGs are mainly involved in functions related to “neutrophil activation”, “myeloid leukocyte migration”, “neutrophil chemotaxis”, “neutrophil migration”, “immune response-regulating signaling pathway” and “cytokine mediated signaling pathway” (**Figure 2A**); KEGG shows that these DEGs related signaling pathways including “neutrophil extracellular trap formation”, “TNF signaling pathway”, “Th17 cell differentiation”, “Apoptosis”, “IL-17 signaling pathway”, and “Leukocyte transendothelial migration” (**Figure 2B**). As a result, these DEGs have a close connection to NETosis. To identify key genes among these DEGs, we intersected them with DEGs between normal tissues and LUAD in bulk datasets, and also with genes whose *P-*value in univariate cox regression were less than 0.04, and finally acquired 61 genes (**Figure 2C**). Most of the 61 genes were positively correlated, but some were negatively correlated, according to the correlation heatmap (**Figure 2D**). Next, we observed that the clustering effect was most optimal when k=2 (**Figure 2E**), allowing us to group all LUAD patients into two clusters based on these 61 genes. Most of these 61 genes exhibited high expression in cluster 1 (**Figure 2F**), which was associated with significantly better prognosis (**Figure 2G**), earlier stages, and lower mortality events (**Figure 2H**) compared to cluster 2. Furthermore, ssGSEA analysis indicated that cluster 1 had significantly higher infiltration of immune cells compared to cluster 2 (**Figure 2I**). Therefore, patients in these two NETosis clusters exhibited distinct characteristics.

### Construction and validation of the NETosis-related Riskscore

**Figure 3:**
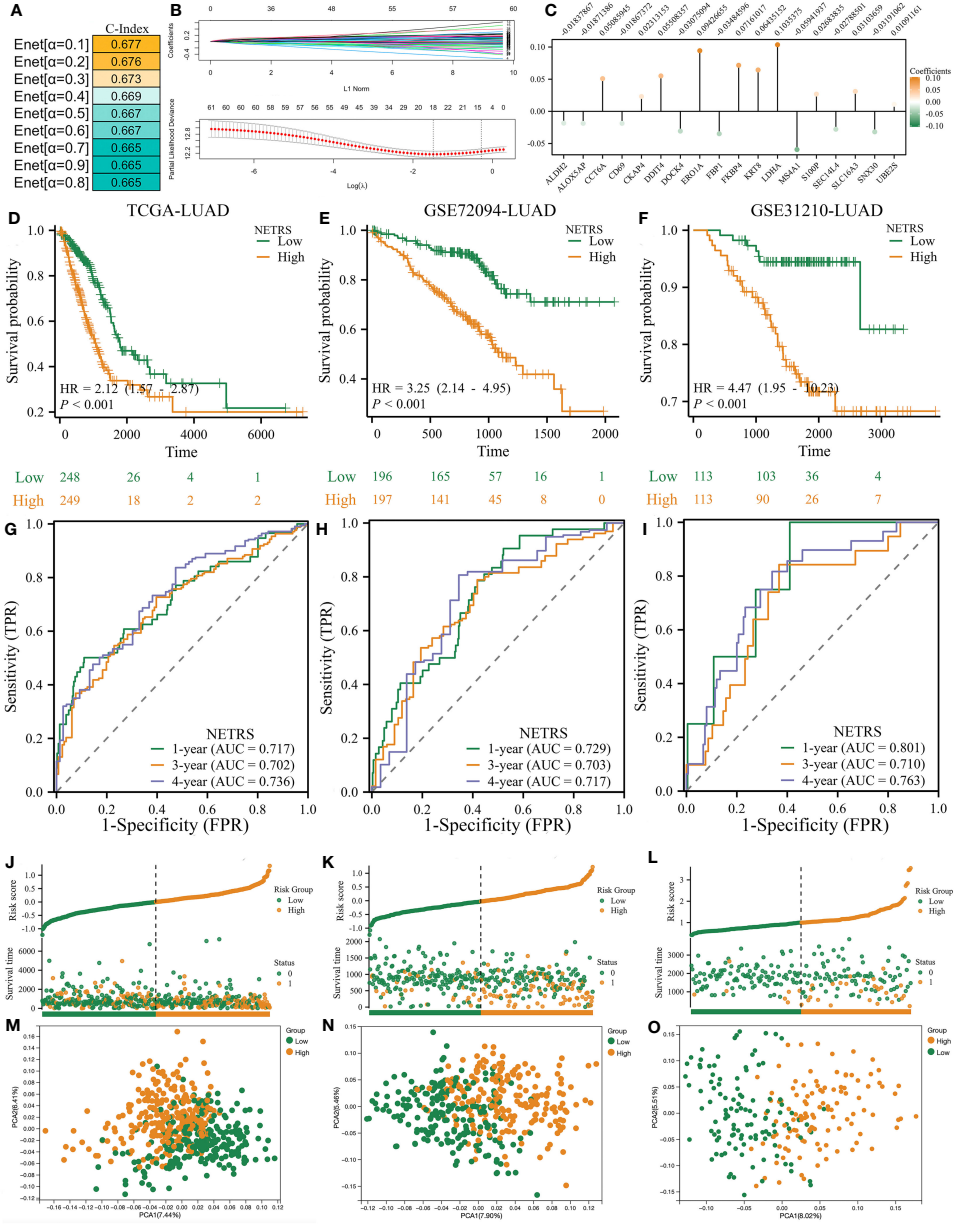
The NETRS was identified and confirmed. **(A)** When setting the α value as 0.1, the Enet model gets its highest C-index. **(B, C)** The Enet algorithm (α=0.1) identified 18 NETosis-related genes **(B)** and their corresponding coefficients **(C)**. **(D–F)** Patients’ different prognosis between high- and Low- NETRS groups in TCGA **(D)**, GSE72094 **(E)**, and GSE31210 **(F)** sets. **(G–I)** The time-ROC curves shows the AUC value of NETRS in predicting patients’ survival in TCGA **(G)**, GSE72094 **(H)**, and GSE31210 **(I)** sets. **(J–L)** Patients’ different OS events between high- and low- NETRS groups in TCGA **(J)**, GSE72094 **(K)**, and GSE31210 **(L)** sets. **(M–O)** The PCA analysis showed that two NETRS group patients’ characteristics differed extinct in the TCGA **(M)**, GSE72094 **(N)**, and GSE31210 () sets.


To better predict patients’ survival based on NETosis-related genes, we performed Elastic Network on these 61 prognostic related genes. By utilizing the TCGA-LUAD as the training dataset, it was observed that the prognostic model achieved its highest C-Index when the α value was set to 0.1 (**Figure 3A**). Therefore, we obtain a NETosis-Related Riskscore (NETRS) consisting of 18 NETosis-Related Genes (**Figure 3B**) and their corresponding coefficients (**Figure 3C**) by the Enet algorithm (α=0.1).

$$\begin{array}{l} NETRS=\text{ALDH}2*(-0.01837867)+\text{ALOX}5\text{AP}*(-0.01871386)+\text{CCT}6\text{A}*\\ (0.05085945)+\text{CD}69*(-0.01867372)+\text{CKAP}4*(0.02313153)+\text{DDIT}4*\\ (0.05508357)+\text{DOCK}4*(-0.03075094)+\text{ERO}1\text{A}*(0.09426655)+\text{FBP}1*\\ (-0.03484596)+\text{FKBP}4*(0.07161017)+\text{KRT}8*(0.06435152)+\text{LDHA}*\\ (0.10353750)+\text{MS}4\text{A}1*(-0.05941937)+\text{S}100\text{P}*(0.02683835)+\text{SEC}14\text{L}4*\\ (-0.02788501)+\text{SLC}16\text{A}3*(0.03103659)+\text{SNX}30*(-0.03191062)+\text{UBE}2\text{S}*\\ (0.01091161) \end{array}$$

The median NETRS value was used to categorize patients into two groups. This showed that patients in the high-NETRS group had a significantly worse prognosis than patients in the low-NETRS group, not only in the training set TCGA-LUAD (**Figure 3D**), but also in two external validation sets, namely GSE72094-LUAD (**Figure 3E**), and GSE31210-LUAD (**Figure 3F**). Based on the time-ROC curves, NETRS was able to predict patients' prognoses at 1, 3, and 4 years with an AUC value greater than 0.7, indicating satisfactory predictive ability (**Figure 3G-I**). Additionally, patients with high NETRS experienced a higher rate of mortality (**Figure 3J-L**), and the PCA analysis revealed noticeable differences between patients with a high and low NETRS (**Figure 3M-O**).

### NETRS strongly correlates with LUAD patients’ clinical characteristics

**Figure 4:**
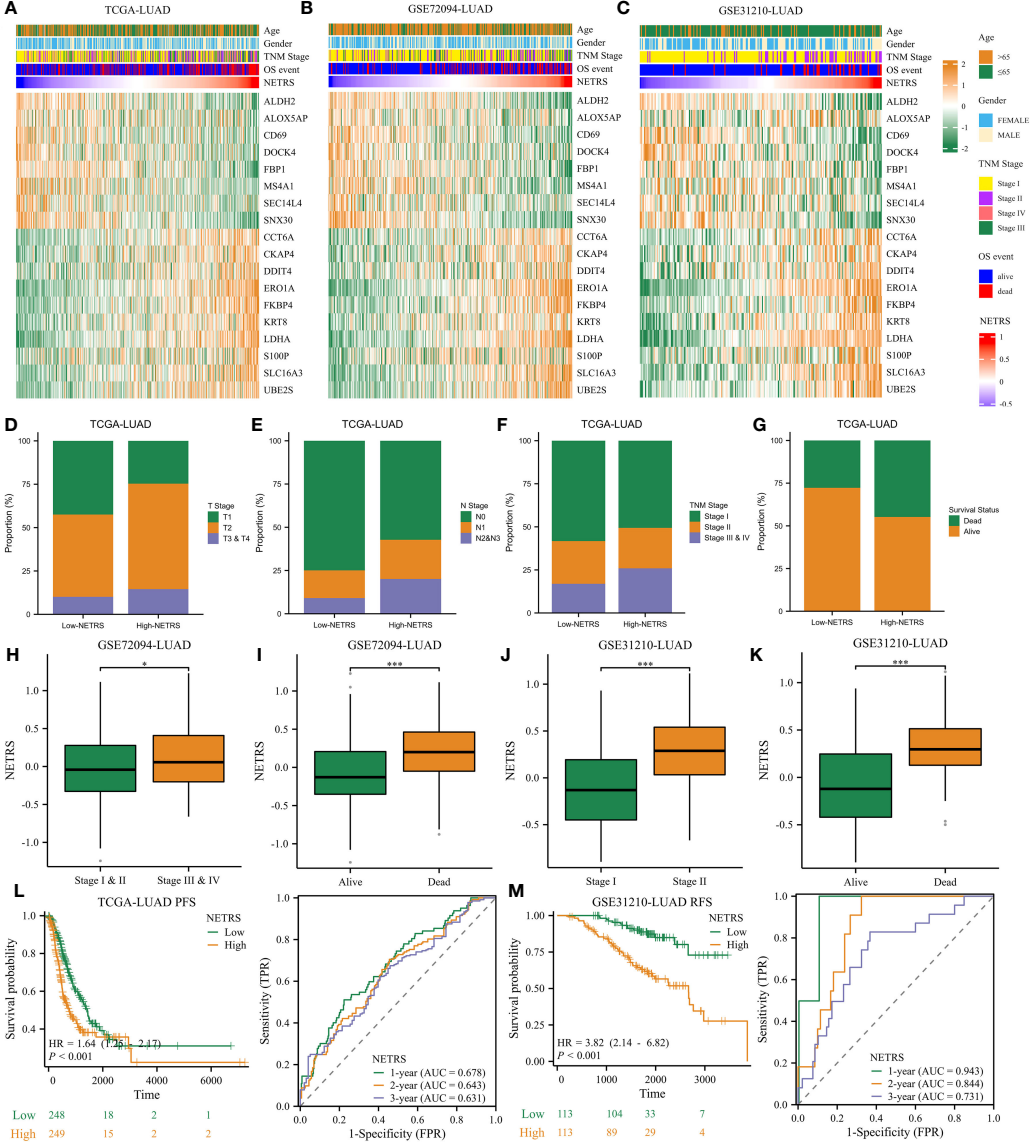
NETRS strongly correlates with LUAD patients’ clinical features. **(A–C)** Heatmaps showed the expression pattern of the 18 genes that make up NETRS in the TCGA **(A)**, GSE72094 **(B)**, and GSE31210 **(C)** sets. **(D–G)** LUAD patients in the high-NETRS group exhibited higher proportion of advanced T **(D)**, N **(E)**, pathologic Stage **(F)**, and lethal OS event **(G)** in the TCGA-LUAD cohort. **(H, I)** In GSE72094 cohort, patients’ NETRS increased with Stage progression **(H)** and lethal OS event **(I)**. **(J, K)** In GSE31210 cohort, patients’ NETRS increased with Stage progression **(J)** and lethal OS event **(K)**. **(L)** NETRS’s efficacy in predicting LUAD patients’ PFS in TCGA cohort. **(M)** NETRS’s efficacy in predicting LUAD patients’ RFS in GSE31210 cohort. ‘*’ means P value is less than 0.05, ‘***’ means P value is less than 0.001.

The heatmap illustrates the expression patterns of the 18 genes comprising NETRS in the TCGA (**Figure 4A**), GSE72094 (**Figure 4B**), and GSE31210 (**Figure 4C**) datasets. We observed that in all datasets, ALDH2, ALOX5AP, CD69, DOCK4, FBP1, MS4A1, SEC14L4, and SNX30 were highly expressed in the low-NETRS group, while the remaining 10 genes were highly expressed in the high-NETRS group. In the TCGA-LUAD cohort, patients in the high-NETRS group exhibited a higher proportion of advanced T Stage (**Figure 4D**), N Stage (**Figure 4E**), Clinical Stage (**Figure 4F**), and deceased Survival status (**Figure 4G**). Furthermore, in the GSE72094 and GSE31210 cohorts, NETRS also increased with advanced stages (**Figures 4H, J**) and deceased status (**Figures 4I, K**). Additionally, we discovered that in the TCGA cohort, patients’ Progress Free Survival (PFS) decreased as their NETRS increased (**Figure 4L**). Similarly, in the GSE31210 cohort, patients’ Relapse Free Survival (RFS) followed a similar trend (**Figure 4M**). Surprisingly, in the GSE31210 cohort, NETRS achieved an impressive predictive power for patients’ 1-year RFS, with a score of 0.943 (**Figure 4M**), highlighting its excellent predictive capability.

### Comparison of NETRS with previously published prognostic models

**Figure 5:**
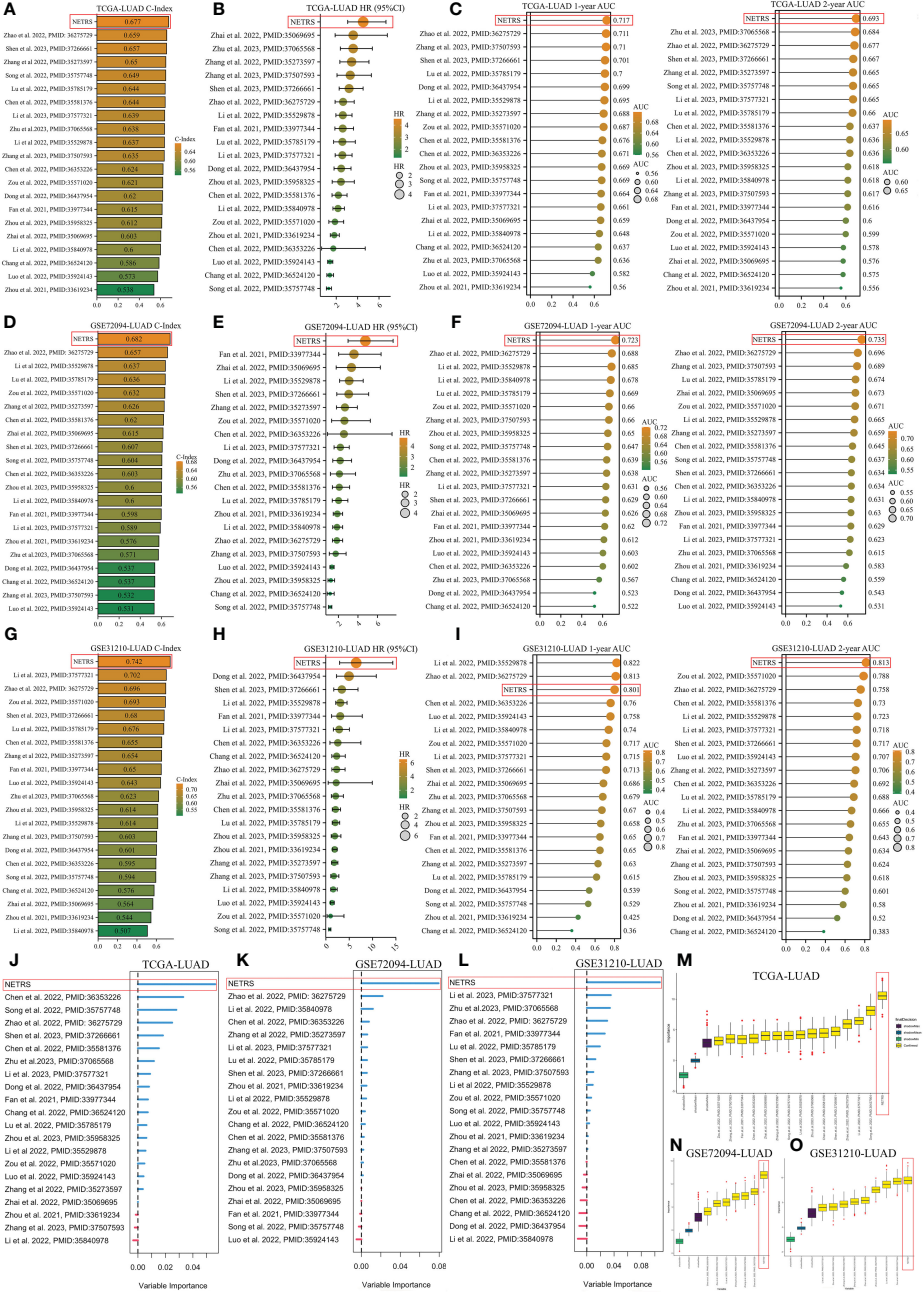
Comparing NETRS with previously published 20 gene signatures**. (A, D, G)** Comparison of the C-Index of NETRS and 20 gene signatures in TCGA **(A)**, GSE72094 **(D)**, and GSE31210 **(G)** cohorts. **(B, E, H)** Comparison of the HR value of NETRS and 20 gene signatures in TCGA **(B)**, GSE72094 **(E)**, and GSE31210 **(H)** cohorts. **(C, F, I)** Comparison of the AUC value of NETRS and 20 gene signatures in TCGA **(C)**, GSE72094 **(F)**, and GSE31210 **(I)** cohorts. **(J–L)** Exploring the importance of the 21 gene signatures in predicting patients’ survival in TCGA **(J)**, GSE72094 **(K)**, and GSE31210 **(L)** cohorts using RandomForest. **(M–O)** Exploring the importance of the 21 gene signatures in predicting patients’ survival in TCGA **(M)**, GSE72094 **(N)**, and GSE31210 **(O)** cohorts using Boruta.

In order to demonstrate the superiority of NETRS, we compared its predictive power with 20 previously published prognostic gene signatures for LUAD. These signatures consisted of functional genes related to lactic acid metabolism (PMID:36275729), mitotic spindle (PMID:37266661), autophagy (PMID:35529878), inflammation (PMID:35069695), cuproptosis (PMID:36353226), pyroptosis (PMID:36437954), and apoptosis (PMID:35571020). Genes’ coefficients and calculation formulas for each signature can be found in the respective articles. Then we extracted the gene signatures from the found candidate studies and applied them to our three study cohorts (TCGA-LUAD, GSE72094-LUAD, and GSE31210-LUAD). By calculating the C-Index for each gene signature, it was observed that NETRS exhibited the highest C-Index among the three cohorts (**Figures 5A, D, G**). Univariate Cox regression analysis revealed that NETRS exhibited the highest HR value, indicating a higher risk compared to these signatures (**Figures 5B, E, H**). Moreover, in terms of predicting patients’ prognosis at 1- and 2- years, NETRS outperformed almost all signatures, as indicated by the higher AUC value (**Figures 5C, F, I**). In the GSE31210 cohort, NETRS demonstrated slightly weaker performance than Li, Zhao et al.’s signatures in predicting patients’ 1-year survival (**Figure 5I**). However, in other cohorts, the performance of Li, Zhao et al.’s signatures were significantly lower than that of NETRS, suggesting a chance effect. Additionally, by utilizing two machine learning algorithms, namely RandomForest and Boruta, we have further validated the importance of NETRS. Both algorithms consistently ranked NETRS as the most significant feature among all 21 signatures (**Figures 5J–O**). In general, NETRS exhibited superior predictive capabilities for the prognosis of LUAD patients compared to the other 20 gene signatures, thereby establishing itself as a more dependable prognostic indicator.

### Construction and validation of a nomogram

**Figure 6:**
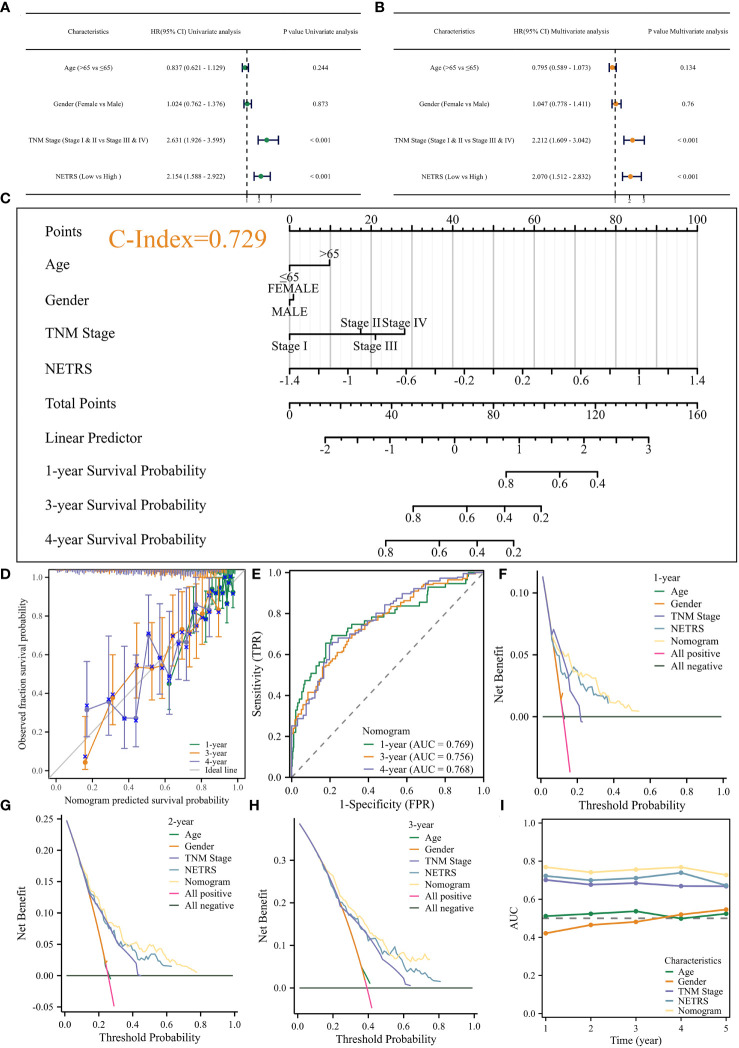
Construction of the Nomogram**. (A, B)** Uni- **(A)** and Multi- **(B)** variable Cox regression analysis identified NETRS as an independent prognostic factor. **(C–E)** The nomogram **(C)** and its calibration **(D)**, and ROC **(E)** curves. **(F–H)** The DCA curves showed that the nomogram has stronger power for predicting patients’ 1- **(F)**, 2- **(G)**, and 3- **(H)** years’ survival than single clinical factors and NETRS. **(I)** Time-AUC curves shows that after incorporating clinical factors, NETRS has stronger power for predicting patients’ survival.

NETRS was evaluated as an independent prognostic factor using univariate and multivariate Cox regression analysis. The results demonstrated that even after accounting for clinical factors, NETRS still significantly impacted the prognosis of LUAD patients. This suggests that NETRS can serve as an independent prognostic indicator, not only in the TCGA-LUAD cohort (**Figures 6A, B**), but also in the GSE72094 (**Table 1**) and GSE31210 (**Table 2**) cohorts. By combining NETRS with clinical factors like TNM Stage, age, and gender, we developed a nomogram to predict the prognosis of LUAD patients. The nomogram had a C-Index of 0.729 in TCGA (**Figure 6C**), and its calibration curve (**Figure 6D**) and time-ROC curve (**Figure 6E**) confirmed its reliable predictive ability. Additionally, the DCA curves indicated that when integrated with clinical characteristics, NETRS exhibited a more robust predictive capability (**Figures 6F–H**). Finally, based on the time-AUC curve, we can conclude that the nomogram had the strongest predictive power, followed by NETRS and TNM Stage, while age and gender had the least predictive impact (**Figure 6I**).

**Table 1:** The NETRS in GSE72094 cohort was analyzed using both univariable and multivariable Cox regression analysis.

Characteristics	Univariate analysis		Multivariate analysis	
	Hazard ratio (95% CI)	P value	Hazard ratio (95% CI)	P value
**Age (**≤65 vs >65)	0.712 (0.464 - 1.093)	0.121	0.697 (0.451 - 1.075)	0.102
**Gender** (female vs male)	1.547 (1.065 - 2.246)	**0.022**	1.623 (1.110 - 2.374)	**0.012**
**TNM Stage** (I&II vs III&IV)	2.607 (1.736 - 3.914)	**< 0.001**	2.655 (1.759 - 4.007)	**< 0.001**
**NETRS** (low vs high)	3.218 (2.114 - 4.899)	**< 0.001**	3.177 (2.082 - 4.849)	**< 0.001**

The bold values means that the values were statistically significant, meaning that the p-value was less than 0.05.

**Table 2:** The NETRS in GSE31210 cohort was analyzed using both univariable and multivariable Cox regression analysis.

Characteristics	Univariate analysis		Multivariate analysis	
	Hazard ratio (95% CI)	P value	Hazard ratio (95% CI)	P value
**Age (**≤65 vs >65)	2.583 (1.313 - 5.083)	**0.006**	4.409 (2.145 - 9.066)	**< 0.001**
**Gender** (female vs male)	1.519 (0.780 - 2.955)	0.219	1.379 (0.703 - 2.707)	0.350
**TNM Stage** (I vs II)	4.232 (2.175 - 8.236)	**< 0.001**	3.226 (1.583 - 6.575)	**0.001**
**NETRS** (low vs high)	7.016 (2.720 - 18.096)	**< 0.001**	5.983 (2.251 - 15.898)	**< 0.001**

The bold values means that the values were statistically significant, meaning that the p-value was less than 0.05.

### Exploring NETRS associated biological functions

**Figure 7:**
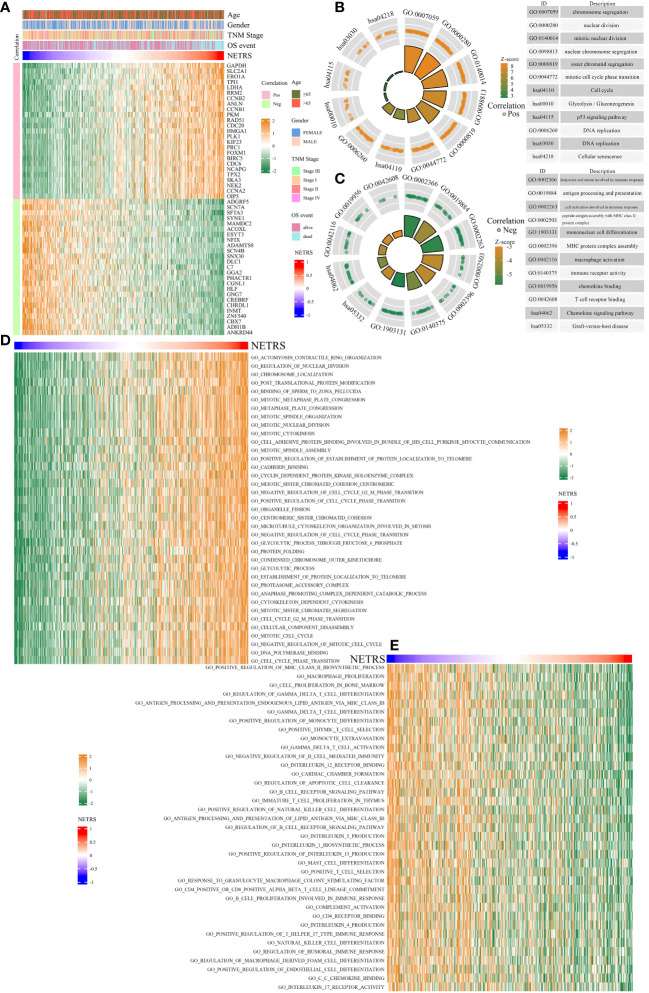
Enrichment analysis of potential biological functions associated with NETRS. **(A)** The heatmap shows the top 50 genes which are most correlated with NETRS. **(B, C)** GO and KEGG analysis showed the functions of genes positively **(B)** or negatively **(C)** correlated with NETRS. **(D, E)** GSVA analysis revealed the gene sets with high activity in the high NETRS group **(D)** and the gene sets with high activity in the low NETRS group **(E)**.

In order to clarify the mechanism behind NETRS’ excellent predictive abilities, further studies were carried out. Correlation analysis was performed to identify genes associated with NETRS, and we visualized the top 50 correlated genes (**Table S2**; **Figure 7A**). Genes positively correlated with NETRS were mainly involved in biological processes, such as ‘Cell cycle’, ‘DNA replication’, ‘chromosome segregation’, ‘nuclear division’, and ‘p53 signaling pathway’ (**Figure 7B**); while genes negatively correlated with NETRS were mainly concentrated in some immune-related functions such as ‘macrophage activation’, ‘immune receptor activity’, ‘chemokine binding’, ‘MHC protein complex assembly’, and ‘mononuclear cell differentiation’ (**Figure 7C**). Besides, cell cycle associated gene sets had higher activity in the high-NETRS group (**Figure 7D**), while immune-function related gene sets scored higher in the low-NETRS group (**Figure 7E**), which echoing the results of GO and KEGG analysis.

### Exploring NETRS at single-cell level

**Figure 8:**
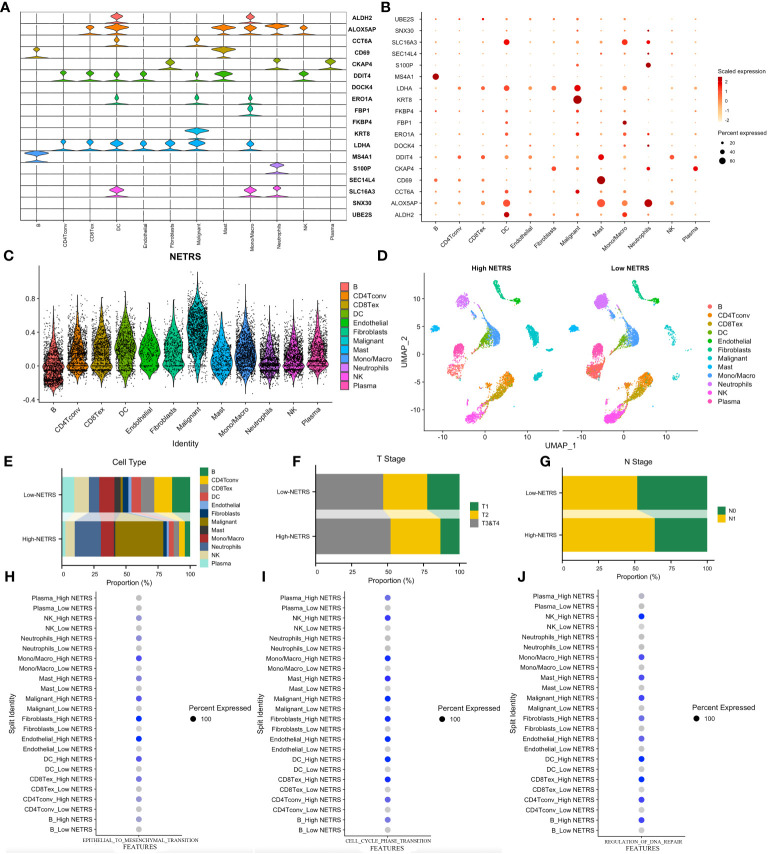
Exploring NETRS’s distribution at cellular level. **(A, B)** The violin plot **(A)** and the dot plot **(B)** showed the 18 NETRS genes’ expression at different cell types. **(C)** Different cells’ NETRS level. **(D)** On the UMAP plot, the distribution of cells in high and low NETRS groups is shown. **(E–G)** Different cell **(E)**, T Stage **(F)**, and N Stage **(G)** proportion at different NETRS groups. **(H–J)** High-NETRS cells had higher EMT **(H)**, Cell cycle **(I)**, and DNA replication activity.

**Figures 8A, B** illustrate the distribution of cellular expression for 18 NETRS genes. It was observed that LDHA was expressed in seven different cell types, MS4A1 was exclusively expressed in B cells, S100P only in neutrophils, and FBP1 exclusively in mono/macrophages. SNX30, UBE2S, SEC14L4, FKBP4, and DOCK4 exhibited low expression levels across all cell types. Moving forward, we performed NETRS calculations at the single-cell level, which revealed that malignant cells exhibited the highest NETRS values (**Figure 8C**). By categorizing all cells into two groups based on the mean NETRS value (**Figure 8D**), we observed that the high NETRS group consisted of a larger proportion of tumor cells (**Figure 8E**), advanced T (**Figure 8F**) and N (**Figure 8G**) stages. Additionally, we validated enrichment analysis results in bulk datasets, demonstrating that cells with high NETRS scores exhibited elevated EMT (**Figure 8H**), cell cycle (**Figure 8I**), and DNA replication repair (**Figure 8J**) scores, indicative of more malignant characteristics.

### NETRS significantly affects the TME

**Figure 9:**
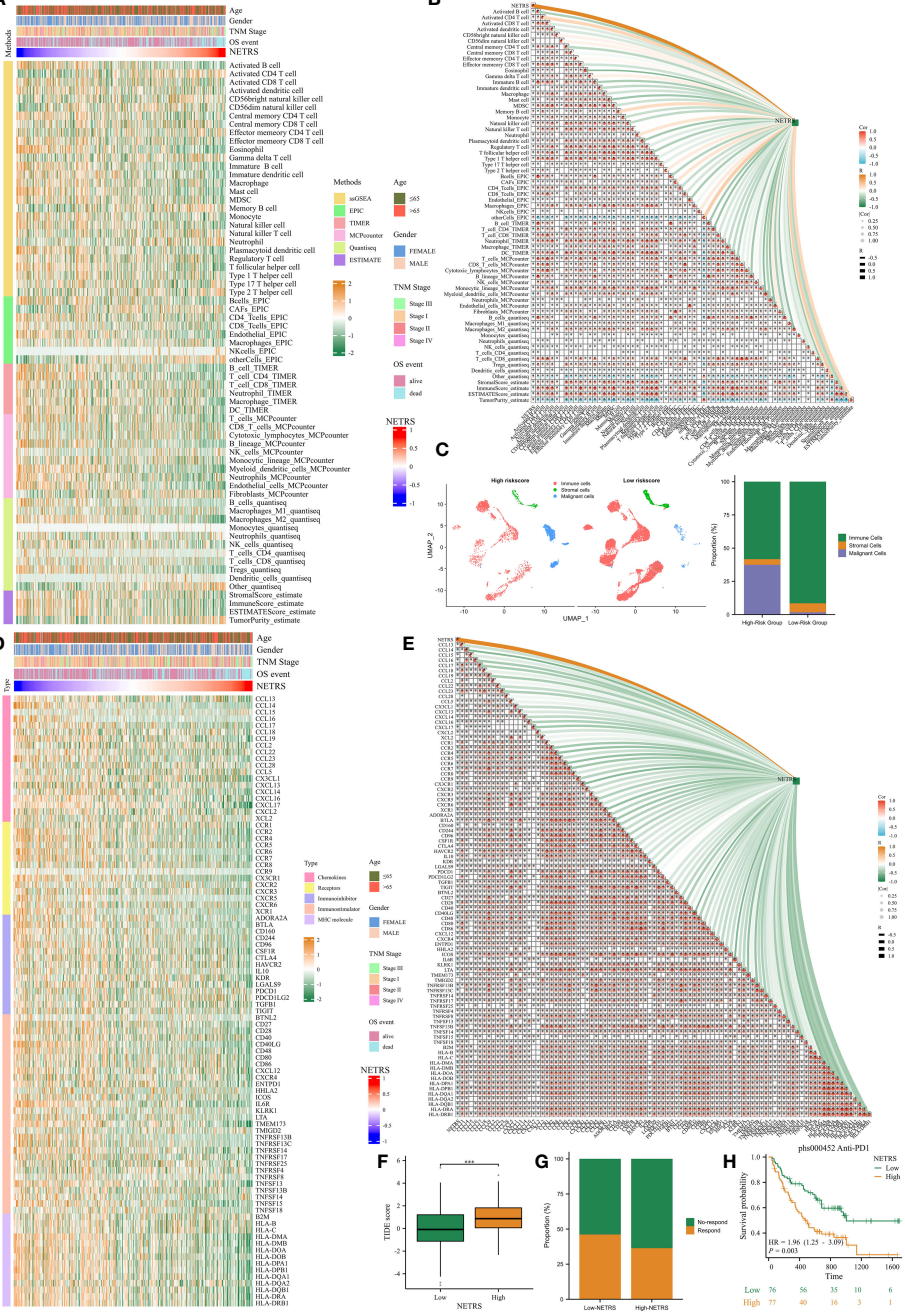
The effects of NETRS on the TME. **(A)** Immunocytes infiltrated significantly different between high- and low- NETRS groups. **(B)** NETRS negatively correlated with immune cells’ infiltration. **(C)** In sc-RNA set, low-NETRS group had higher immune cell level. **(D)** TME modulators expressed significantly different between two NETRS groups. **(E)** NETRS negatively correlated with TME modulators’ expression. **(F)** Patients with low NETRS had lower TIDE score. **(G)** The low-NETRS group had higher ICB responders’ proportion. **(H)** Patients receiving immunotherapy with lower NETRS had better prognosis. ‘***’ means P value is less than 0.001.

Due to the immune-hot characteristics observed in patients with low NETRS (**Figure 7E**), we conducted a comprehensive investigation into the relationship between NETRS and the tumor microenvironment (TME). After verifying six algorithms, we found that the low-NETRS group exhibited higher levels of immune cell infiltration (**Figure 9A**). This pattern was also observed in the single-cell dataset (**Figure 9C**). Furthermore, the low-NETRS group displayed significantly higher expression of various TME regulators, including immunoinhibitors, stimulators, chemokines, and MHC molecules (**Figure 9D**). Remarkably, we observed a significant positive correlation between most immune cells, while a significant negative correlation was evident between NETRS and the majority of immune cells (**Figure 9B**). Similar correlations were observed among TME regulators, with NETRS demonstrating significant negative associations with most of them (**Figure 9E**). Additionally, patients with low NETRS showed a marked reduction in the TIDE score, indicating a potentially greater benefit from immune checkpoint blockade (ICB) therapy for these individuals (**Figure 9F**). This hypothesis was validated in the ICB therapy cohort phs000452, where patients with low NETRS exhibited a higher proportion of ICB responders (**Figure 9G**) and showed improved prognosis (**Figure 9H**).

### Multi-omics comparison between NETRS-high and NETRS-low groups in TCGA- LUAD

**Figure 10:**
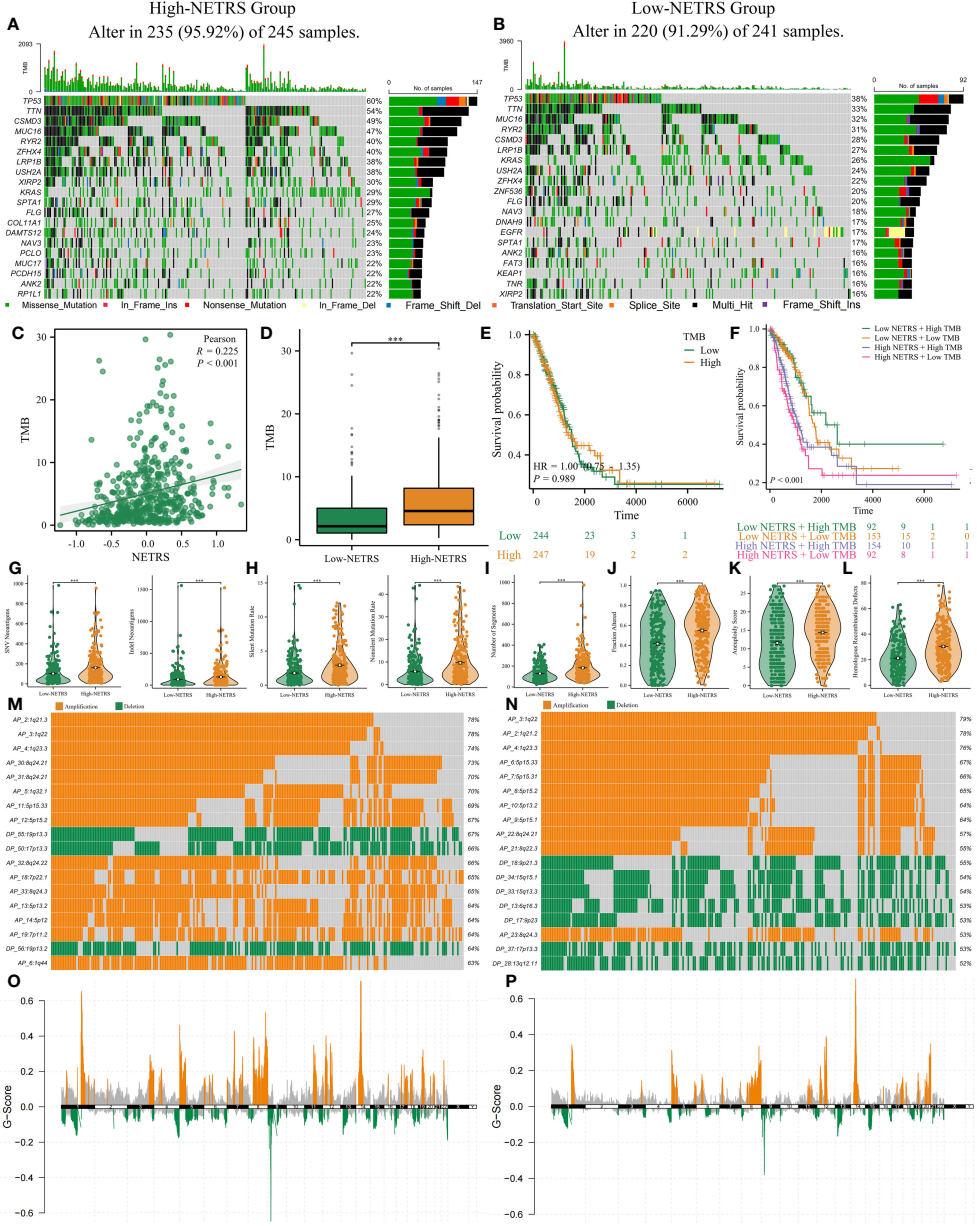
The multi-omics landscape differences between high and low NETRS groups**. (A, B)** High- **(A)** and low- **(B)** NETRS patients’ somatic mutation frequency. **(C, D)** NETRS’s correlation with TMB **(C)** and different NETRS groups patients’ TMB difference **(D)**. **(E, F)** TMB **(E)** or TMB combined with NETRS’s effect **(F)** on patients’ survival. **(G–L)** High- and low- NETRS group patients’ different neoantigen load **(G)**, gene mutation rate **(H)**, number of segments **(I)**, fraction altered **(J)**, aneuploidy score **(K)**, and homologous recombination defect **(L)**. **(M, N)** The top 18 CNV events in high- **(M)** and low- **(N)** NETRS groups. **(O, P)** ChromPlots show patients’ G-score in high- **(O)** and low- **(P)** NETRS groups. ‘***’ means P value is less than 0.001.

The top 20 genes with the highest mutation frequency were analyzed and visualized between groups with high and low NETRS scores (**Figures 10A, B**). Patients in the high-NETRS group had significantly higher gene mutation frequency (high-NETRS: TP53:60%, TTN: 54%, CSMD3: 49%, MUC16: 47%, RYR2: 40%; low-NETRS: TP53: 38%, TTN: 33%, CSMD3: 28%, MUC16: 32%, RYR2: 31%). Besides, NETRS posotively correlated with TMB (**Figure 10C**), and patients in high-NETRS group had higher TMB (**Figure 10D**). Survival analysis showed that TMB didn’t affect patients’ prognosis (**Figure 10E**). However, after combining TMB with NETRS, it can better stratify patients’ survival (**Figure 10F**), and patients with Low NETRS + High TMB had relatively best prognosis. Patients in high-NETRS group had higher neoantigen load (**Figure 10G**), higher gene mutation rate (**Figure 10H**), high number of segments (**Figure 10I**), fraction altered (**Figure 10J**), aneuploidy score (**Figure 10K**), and homologous recombination defect (**Figure 10L**). Besides, we found that the CNV event also differed significantly between two NETRS groups (**Figures 10M, N**). Patients in the high-NETRS group had a higher frequency of CNV event, and most of them were amplification; while patients in the low-NETRS group had relatively lower frequency of CNV, and deletion had higher proportion. In addition, we found from the ChromPlots that patients in the high NETRS group had significantly higher G-Scores than patients in the low NETRS group (**Figures 10O, P**). Thus, high-NETRS patients with LUAD were more likely to display malignant characteristics.

### Identification of potential drugs targeting NETRS

**Figure 11:**
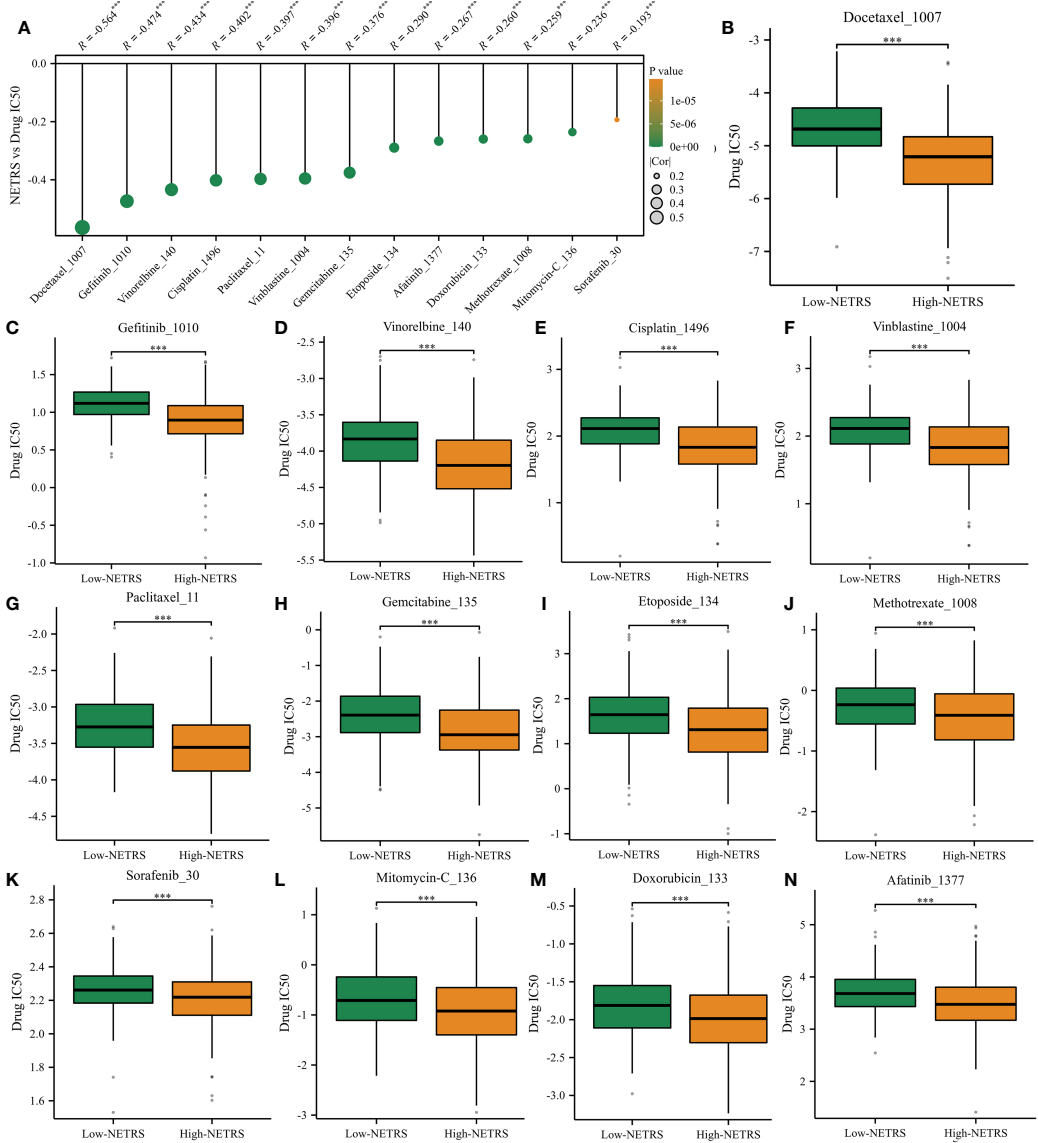
High-NETRS patients may more sensitivity to chemotherapy. **(A)** NETRS’s relationship with drugs’ IC50 value. **(B–N)** Comparison of the IC50 value of Docetaxel **(B)**, Gefitinib **(C)**, Vinorelbine **(D)**, Cisplatin **(E)**, Vinblastine **(F)**, Paclitaxel **(G)**, Gemcitabine **(H)**, Etoposide **(I)**, Methotrexate **(J)**, Sorafenib **(K)**, Mitomycin-C **(L)**, Doxorubicin, **(M)** and Afatinib **(N)** between high- and low- NETRS groups’ patients.

Based on the drug information obtained from GDSC1, we investigated the relationship between NETRS and commonly used drugs for the clinical treatment of LUAD. Through correlation analysis, we found a negative correlation between NETRS and the IC50 values of 13 drugs (**Figure 11A**). These drugs, namely Docetaxel, Gefitinib, Vinorelbine, Cisplatin, Vinblastine, Paclitaxel, Gemcitabine, Etoposide, Methotrexate, Sorafenib, Mitomycin-C, Doxorubicin, and Afatinib, exhibited significantly lower IC50 values in the high-NETRS group compared to the low-NETRS group. This suggests that patients in the high-NETRS group may potentially benefit more from these drugs (**Figures 11B–N**).

### NETRS is also valuable in predicting all NSCLC patients’ survival

**Figure 12:**
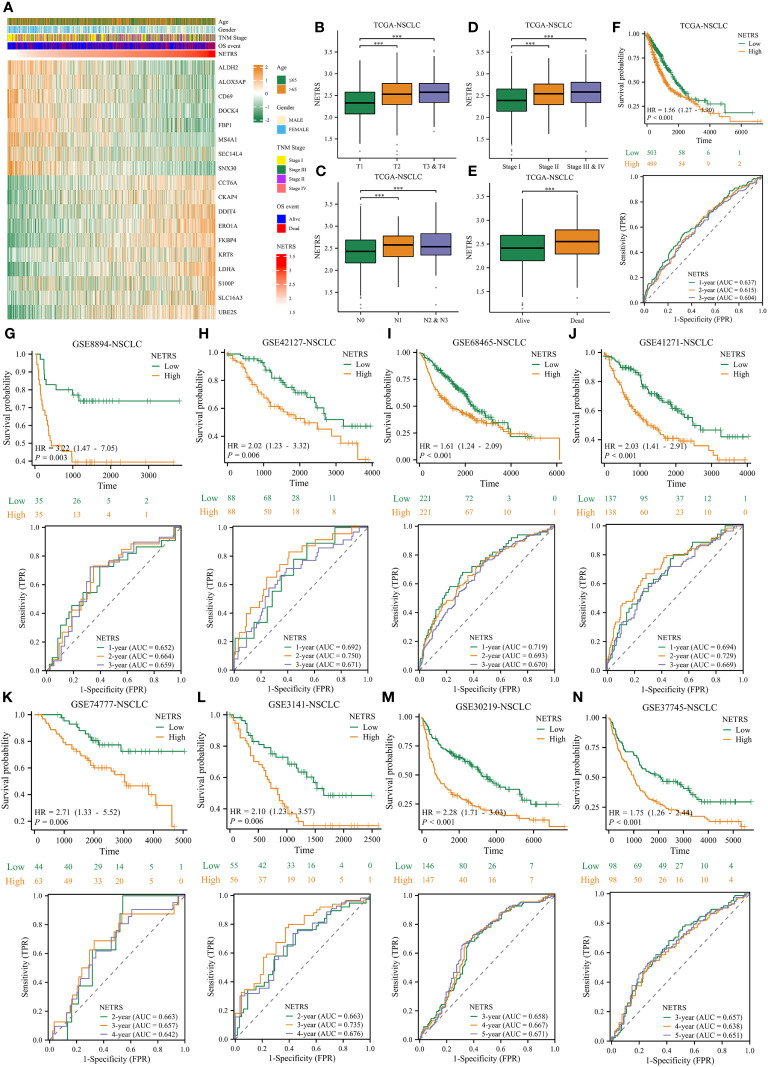
The value of NETRS in predicting the prognosis of NSCLC patients. **(A)** The 13 NETRS genes’ expression pattern in TCGA-NSCLC cohort. **(B–E)** NSCLC patients’ NETRS between different T **(B)**, N **(C)**, TNM **(D)** Stages, and survival events **(E)**. **(F–N)** The prognosis of NSCLC patients among high- and low- NETRS groups at TCGA-NSCLC **(F)**, GSE8894 **(G)**, GSE42127 **(H)**, GSE68465 **(I)**, GSE41271 **(J)**, GSE74777 **(K)**, 3141 **(L)**, GSE30219 **(M)**, and GSE307745 **(N)** sets and the corresponding time-dependent ROC curves. ‘***’ means P value is less than 0.001.

In light of these analyses, NETRS is a superior and robust prognosticator for patients with LUAD. However, NSCLC includes many subtypes, including LUAD, LUSC (lung squamous cell carcinoma), lung large cell carcinoma, lung basal cell carcinoma et al. Aiming to assess how well NETRS predicts the prognosis of patients with all forms of NSCLC, we collected nine independent datasets of NSCLC patients. In these nine datasets, except for the GSE74777 dataset only includes data for LUSC patients, all datasets contain data for patients with various NSCLC types. **Figure 12A** shows the expression of 18 NETRS genes in the TCGA-NSCLC cohort. In this cohort, NETRS increased with advanced T (**Figure 12B**), N (**Figure 12C**), and TNM stages (**Figure 12D**), and dead survival event (**Figure 12E**), similar to the results in LUAD cohorts. Including the TCGA-NSCLC cohort, nine independent cohorts found that NSCLC patients with high NETRS had a significantly worse prognosis than those with low NETRS (all *P*-value < 0.01), and the ROC curves demonstrated that NETRS is highly predictive (**Figures 12F–N**). Therefore, NETRS is also valuable in predicting the prognosis of patients suffering from all types of NSCLC.

### Validation of six NRGs’ expression using three datasets, qRT-PCR, and Immunohistochemical staining

**Figure 13:**
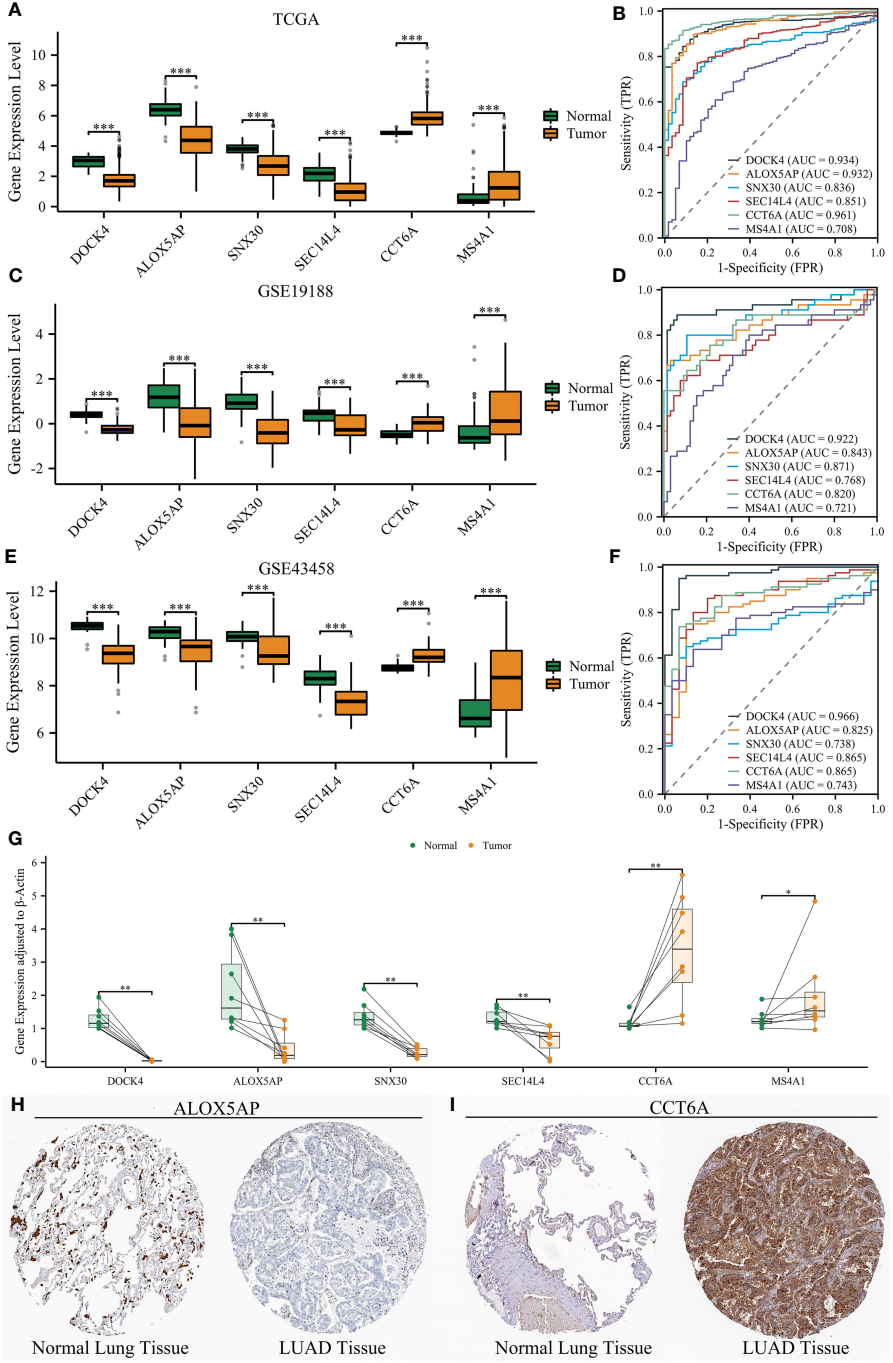
Validation of six NRGs’ expression using three datasets and clinical samples. **(A, B)** Six NRGs’ differential expression between normal lung and LUAD tissues **(A)**, and their diagnostic value **(B)** in the TCGA dataset. **(C, D)** Six NRGs’ differential expression between normal lung and LUAD tissues **(C)**, and their diagnostic value **(D)** in the GSE19188 dataset. **(E, F)** Six NRGs’ differential expression between normal lung and LUAD tissues **(E)**, and their diagnostic value **(F)** in the GSE43458 dataset. **(G)** The differential expression of six NRGs between normal lung and LUAD tissues by qRT-PCR analysis. **(H–I)** Immunohistochemical staining confirmed the decreased protein level of ALOX5AP **(H)** and increased protein level of CCT6A **(I)** in LUAD tissues. ‘*’ means P value is less than 0.05, ‘**’ means P value is less than 0.01, and ‘***’ means P value is less than 0.001.

NETRS is comprised of 18 NRGs, of which six genes - ALOX5AP, DOCK4, CCT6A, MS4A1, SEC14L4, and SNX30 - have been minimally investigated in relation to LUAD. To further explore the potential involvement of the six NRGs in LUAD, we conducted a comparative analysis of their expression levels between LUAD and normal tissues. In three separate datasets, we observed high expression levels of DOCK4, ALOX5AP, SNX30, and SEC14L4 in normal lung tissues. Conversely, LUAD tissues showed high expression levels of CCT6A and MS4A1 (**Figures 13A, C, E**). Furthermore, the ROC curves demonstrated that all the six NRGs exhibited high diagnostic accuracy (**Figures 13B, D, F**).

Although we have unveiled the differential expression of the six NRGs, all the analyses were performed using publicly available databases. To further validate the expression of six NRGs and enhance the credibility of NETRS, we collected clinical samples and conducted qRT-PCR analysis. In line with the findings from publicly available databases, DOCK4, ALOX5AP, SNX30, and SEC14L4 exhibited high expression levels in normal lung tissues, while CCT6A and MS4A1 were highly expressed in LUAD tissues (**Figure 13G**). Finally, we selected ALOX5AP and CCT6A as representatives and validated their localization and protein expression in tissues by immunohistochemical staining. Consistent with the previous findings, immunohistochemical staining images demonstrated that ALOX5AP expression was decreased in LUAD (**Figure 13H**), whereas CCT6A expression was increased in LUAD (**Figure 13I**).

## Discussion

There has been considerable progress in treating lung cancer, but it remains a very challenging tumor for medical professionals to treat adequately. The development of high-throughput sequencing technology has led to the discovery of more and more prognostic markers. The role of A newly detected type of programmed cell death is NETosis. NETosis in various tumor pathology is now well established, however it remains unclear what their precise molecular mechanism is (32, 33). This study comprehensively investigated NETosis-related genes in NSCLC due to the limited research on the role of NETosis in this context.

In the first step, the AUCell algorithm was used to calculate the NETosis scores for various cell types. Our results indicated that neutrophils had the highest NETosis score, it may due to the fact that NETosis is a neutrophil-mediated programmed cell death. In the next step, we divided each population into two groups based on their mean NETosis score. According to cell-cell communication analysis, cells with high NETosis Score communicate more frequently and strongly than those with low NETosis Score. This suggests that cells with a high NETosis score may be more active in the TME, leading to anti-disease or pathogenic mechanisms. We then determined DEGs between high NETRS and low NETRS cells, and enrichment analysis showed that these genes were significantly correlated with NETosis. By intersecting DEGs between high and low NETRS cell groups in single-cell dataset, with DEGs between normal tissues and LUAD in bulk datasets, and also with prognostic related genes, 61 NETosis-related Genes (NRGs) were identified. Then based on these 61 NRGs, we identified two NETosis-related clusters. Patients in cluster1 had better prognosis and more active TME characteristics, and most of the 61 NRGs were highly expressed in cluster1, which suggested that cluster1 may more like to be a NETosis-like cluster. Cluster1 also demonstrated a significant improvement in prognosis, suggesting that higher NET activity in LUAD patients’ TME may benefit tumor cell clearance. After that, the NETRS comprised of 18 NRGS was obtained using the Enet algorithm (α=0.1). NETRS proved superior predictive efficacy and was an independent prognostic factor in both training TCGA-LUAD and test sets GSE72094-LUAD and GSE31210-LUAD. Additionally, significant differences were observed between patients categorized into high and low NETRS groups, as determined by PCA analysis. Further exploration was conducted to investigate the potential prognostic mechanism of NETRS, along with the disparities in immune cell infiltration, expression of immune-related molecules, SNV and CNV frequencies, TMB, Neoantigen load, and response to immunotherapy, and drug sensitivity among patients with high and low NETRS. The construction of nomograms revealed that the prognostic ability of NETRS was improved when integrated with clinical parameters, offering a novel approach to predict the prognosis of patients with LUAD. NETRS can also predict all NSCLC patients’ prognosis, which is its another valuable feature: Among more than 2,000 NSCLC patients in nine independent cohorts, patients with high NETRS all had significantly worse outcomes than those with low NETRS, which demonstrated the multifaceted value of NETRS. Lastly, the differential expression of the six identified NRGs was verified using three independent datasets, along with clinical samples gathered from our own collection. These six NRGs have been relatively understudied in LUAD. This study is the first to confirm their expression patterns. Specifically, DOCK4, ALOX5AP, SNX30, and SEC14L4 showed elevated expression levels in normal lung tissues, while CCT6A and MS4A1 exhibited high expression in LUAD tissues. Therefore, this finding not only provides a basis for future investigations on these NRGs in LUAD, but also reinforces the efficacy and reliability of NETRS.

NETRS consisted of 18 NRGs: ALDH2, ALOX5AP, CCT6A, CD69, CKAP4, DDIT4, DOCK4, ERO1A, FBP1, FKBP4, KRT8, LDHA, MS4A1, S100P, SEC14L4, SLC16A3, SNX30, and UBE2S. The lasso regression coefficient of UBE2S, CKAP4, S100P, SLC16A3, CCT6A, DDIT4, KRT8, FKBP4, ERO1A, and LDHA is greater than 0, which were risk factors; while the regression coefficients of MS4A1, FBP1, SNX30, DOCK4, SEC14L4, ALOX5AP, CD69, and ALDH2 were less than 0, which were protective factors. Some of these genes have been extensively studied in relation to LUAD. Overexpression of ALDH2 (protective factor in our study) decreased migration, and proliferation in LUAD cells, but knockdown of ALDH2 increased these properties (34); the growth rate of lung cancer cells overexpressed with CKAP4 (risk factor in our study) was increased *in vivo*, while an antibody against the protein inhibited it (35); LUAD cells were impaired in their ability to invade, metastasize, and proliferate after FBP1 (protective factor in our study) was overexpressed (36); FKBP4 and S100P (risk factors in our study) promotes proliferation and migration of NSCLC cells and inhibits apoptosis, while promoting tumor growth *in vivo* (37, 38). Additionally, GO and GSVA enrichment analyses showed that genes positively associated with NETRS were significantly enriched in functions related to cell cycle. Studies shown that when EOR1A (risk factor in our study, also known as ERO1L) was depleted from NSCLC cells, the expression of factors associated with cell cycle is dramatically reduced (39). Thus, cell cycle-related functions enriched in patients with a high NETRS may as a result of these risky factors such as ERO1A. Besides, enrichment analysis also showed that genes negatively correlated with NETRS were distinctly enriched in functions related to the immune system. We found that CD69 (protective factor in our study) was highly expressed in low-NETRS groups. CD69 has been shown to play an important role in regulating TME, and it may play a role in affecting PD-1 treatment response (40). Thus, immune-related functions enriched in patients with a low NETRS may as a result of these protective factors such as CD69.

Cancer immunotherapy offers new hope to patients with cancerous growths, but evading the immune system remains a formidable challenge to treatment (41, 42). According to our research, individuals belonging to the low NETRS category exhibited elevated levels of immune cell infiltration, increased expression of immune-related substances, including well-known immune checkpoints like PDCD1, BTLA, CTLA4, along with chemokines/receptors and MHC molecules. Consequently, individuals with low levels of NETRS experience ‘immune hot’ symptoms accompanied by greater immune cell infiltration. Moreover, it was discovered that individuals possessing a low NETRS exhibited considerably reduced TIDE scores, which means that the TIDE algorithm anticipated that patients with a low NETRS would display heightened responsiveness to immunotherapy. This hypothesis was confirmed in the phs000452 immunotherapy cohort. Furthermore, we compared the sensitivity of patients in different NETRS groups to a number of drugs commonly used to treat NSCLC in clinics. These was a number of chemotherapy agents can effectively treat patients with NSCLC, such as paclitaxel, docetaxel, cisplatin, vincristine, and vinorelbine. Interestingly, these chemotherapy agents’ IC50 value in patients with high-NETRS patients, which means patients with higher NETRS may more sensitive to these drugs. In addition, the IC50 value of gefitinib and afatinib, which target NSCLC patients with EGFR mutation, remain low in patients with high NETRS (43, 44). Thus, patients with high NETRS would likely benefit more from targeted and chemotherapy therapies, while those with low NETRS would benefit more from immunotherapy.

In spite of the fact that NETosis is a PCD mode which strongly associated with neutrophils, this is the first study to investigate how NETosis-related genesets are expressed by the cells of LUAD tissues, and confirmed that neutrophils has the highest NETosis score in LUAD. Through this, we gained a deeper understanding of NETosis, and provided some novel insights into the effects of NETosis on TME for future studies. This study is also the first to systematically investigate the prognostic role of NETosis-related genes in NSCLC, and the NETRS consisting of 18 NRGs demonstrated its powerful predictive power in 12 cohorts of nearly 3,300 NSCLC patients. In comparison to 20 previously published gene signatures, NETRS had higher C-Index, HR value, and greater accuracy in predicting 1- and 2-year survival of LUAD patients. According to two machine learning algorithms, NETRS had the greatest impact on the survival of LUAD patients compared to these 20 signatures. Thus, we have developed a gene signature that more accurately predicts LUAD prognoses. In addition, we found that when our predecessors applied single-cell sequencing to construct prognostic models, they didn’t combined functional phenotypes, such as the study of Song et al. (PMID:35757748, PMID:35152302) and the study of Zhang et al. (PMID:37507593). In these studies, prognostic models were only constructed based on the markers genes of B cells, NK cells, and DCs, while the relationship between functional phenotypes and cells wasn’t explored. Besides, some studies that targeting functional phenotypes didn’t utilize single-cell sequencing sets, such as the study of Zhao et al. (PMID:36275729) and the study of Li et al. (PMID:35529878). Therefore, we believe that combining single-cell sequencing with functional phenotypes to construct prognostic models can provide new insights into future prognostic studies.

Although the study demonstrated NETRS’s remarkable capability in forecasting prognosis and gauging therapy response in LUAD patients, it still has certain constraints. Initially, all the information in this research originated from publicly accessible databases, and the effectiveness of NETRS was not validated using our own group of subjects. A second reason is that our study only validated the expression patterns of six NRGs and did not extensively investigate their role in LUAD. In our future study, we will confirm the effectiveness of NETRS in larger groups, and additionally investigate the NRGs’ risky or protective role in LUAD further.

To sum up, this research has devised a novel NETRS (NETosis-related Riskscore) which can accurately forecast the prognosis of patients with NSCLC and their reaction treatment. It offers fresh perspectives for future investigations on genes associated with NETosis and the combining use of bulk- and single-cell RNA-sequencing data.

## Data availability statement

The datasets presented in this study can be found in online repositories. The names of the repository/repositories and accession number(s) can be found in the article/**Supplementary Material**.

## Ethics statement

The studies involving humans were approved by The Ethics Committee of the First Affiliate Hospital of Fujian Medical University. The studies were conducted in accordance with the local legislation and institutional requirements. The participants provided their written informed consent to participate in this study.

## Author contributions

LZ: Data curation, Formal Analysis, Investigation, Methodology, Software, Writing – original draft. XZ: Data curation, Investigation, Methodology, Software, Writing – original draft. MG: Data curation, Investigation, Methodology, Software, Writing – original draft. FY: Conceptualization, Supervision, Writing – review & editing. FL: Conceptualization, Supervision, Writing – review & editing.
